# A genome-wide survey of changes in protein evolutionary rates across four closely related species of *Saccharomyces *sensu stricto group

**DOI:** 10.1186/1471-2148-7-9

**Published:** 2007-01-29

**Authors:** Yoshihiro Kawahara, Tadashi Imanishi

**Affiliations:** 1 Integrated Database Group, Biological Information Research Center, National Institute of Advanced Industrial Science and Technology, 2-42 Aomi, Koto-ku, Tokyo 135-0064, Japan; 2 Department of Biological Sciences, Tokyo Metropolitan University, 1-1 Minami-Osawa, Hachioji-shi, Tokyo 192-0397, Japan

## Abstract

**Background:**

Changes in protein evolutionary rates among lineages have been frequently observed during periods of notable phenotypic evolution. It is also known that, following gene duplication and loss, the protein evolutionary rates of genes involved in such events changed because of changes in functional constraints acting on the genes. However, in the evolution of closely related species, excluding the aforementioned situations, the frequency of changes in protein evolutionary rates is still not clear at the genome-wide level. Here we examine the constancy of protein evolutionary rates in the evolution of four closely related species of the *Saccharomyces *sensu stricto group (*S. cerevisiae*, *S. paradoxus*, *S. mikatae *and *S. bayanus*).

**Results:**

For 2,610 unambiguously defined orthologous genes among the four species, we carried out likelihood ratio tests between constant-rate and variable-rate models and found 344 (13.2%) genes showing significant changes in the protein evolutionary rates in at least one lineage. Of all those genes which experienced rate changes, 139 and 49 genes showed accelerated and decelerated evolution, respectively. Most of the evolutionary rate changes could be attributed to changes in selective constraints acting on nonsynonymous sites, independently of species-specific gene duplication and loss. We estimated that the changes in protein evolutionary rates have appeared with a probability of 2.0 × 10^-3 ^per gene per million years in the evolution of the *Saccharomyces *species. Furthermore, we found that the genes which experienced rate acceleration have lower expression levels and weaker codon usage bias than those which experienced rate deceleration.

**Conclusion:**

Changes in protein evolutionary rates possibly occur frequently in the evolution of closely related *Saccharomyces *species. Selection for translational accuracy and efficiency may dominantly affect the variability of protein evolutionary rates.

## Background

The molecular clock hypothesis asserts that the number of amino acid differences in a protein appear to be roughly proportional to the divergence time of the two organisms compared [1,2]. Furthermore, Motoo Kimura claimed that the rate of protein evolution for each protein is approximately constant for various lineages, as long as the function and tertiary structure of the molecule remain essentially unaltered [3]. However, the accuracy of the molecular clock has been controversial for several decades [4,5].

To date, accelerated evolution of some genes in specific lineages has been reported. In particular, changes in the protein evolutionary rates accompanying remarkable phenotypic evolution have been well studied. For example, relative to the lineage leading to rodents, accelerated evolution of the genes involved in the development and physiology of the nervous system was observed in the lineage leading to humans [6]. It was suggested that these genes might have played important roles in the unique evolution of the complex human brain. However, interestingly, some genes involved in the nervous system also showed higher rates of protein evolution in the domesticated dog lineage than in the human lineage, suggesting that the relatively higher rate of evolution in the lineage leading to humans may reflect decelerated evolution in the rodent lineage, or possibly independent adaptive evolution in the human and dog lineages. Thus, it is necessary to be cautious in regarding the accelerated evolution of human nervous system-related genes as representing human-specific innovations [7]. Moreover, using the relative rate test, it has been reported that approximately 0.76% of the proteins seem to have experienced accelerated evolution when comparing across human, mouse and rat [8]. As for other eukaryotic organisms, genome-wide acceleration of protein evolution, which might be caused by physiological and ecological factors that affect the mutation rate, was observed in the diptera lineages when compared with the beetle lineages [9]. According to these studies, in the evolution of morphologically and evolutionarily distant species, the rate of protein evolution seems to change frequently due to adaptive evolution, and changes in mutation rates and selective constraints. Another known cause of changes in protein evolutionary rates is the relaxation of functional constraints acting on duplicate genes following duplication events [10]. In fact, recently duplicated genes evolve faster than unduplicated genes having the same level of divergence and similar functions in 39 genomes from eubacteria, archaea, and eukaryotes [11]. Using amino-acid based method, Zhang et al. [12] found that nearly 60% of duplicated pairs have evolved in an asymmetric divergent manner in the human genome. Analysis of four complete genome sequences (two yeasts, fruit fly and nematode) suggested that 20–30% of duplicate gene pairs show asymmetric evolution that might have been caused by relaxed functional constraints on one of the duplicates [13]. Furthermore, other large-scale studies have shown that duplicate gene sequences often diverge asymmetrically [14,15].

According to the molecular clock hypothesis and aforementioned studies, if the mutation rate and selective constraints on a protein remain constant over time, the rate of protein evolution should also remain constant. However, it is not known whether the protein evolutionary rate is indeed constant in the ordinary state (neither immediately after gene duplication and loss nor during remarkable phenotypic evolution) of the evolution of biologically similar and closely-related species.

Comparative analysis of multiple closely related genomes allows us to accurately trace evolutionary changes at the genome-wide level. Moreover, based on the maximum-parsimony principle and using three or more species for which the phylogenetic relationship is evident, we can estimate the occurrences and the precise directions of evolutionary changes (such as rate acceleration or deceleration) on each lineage. Such an analysis will be feasible for many closely-related species since the genome sequencing projects are complete or in progress for some eukaryotic organisms, such as primates, fruit flies and yeasts [16].

*Saccharomyces cerevisiae*, baker's yeast, is the best-studied eukaryote in terms of molecular cell biology, genetics and genomics. The whole genome sequence of *S. cerevisiae*, the first complete eukaryotic genome sequence, was published in 1996 [17]. The genome sequences of close relatives of *S. cerevisiae *were determined, and more sequencing efforts are still ongoing [16,18,19]. In particular, *S. paradoxus*, *S. mikatae *and *S. bayanus *were frequently used for comparative studies. These species are biologically and morphologically similar to *S. cerevisiae*, and can grow under the same conditions. To date, many researchers have compiled a large amount of biological information for *S. cerevisiae*, such as expression data for each gene [20,21]. Therefore, the use of *S. cerevisiae *as the subject of an evolutionary study would be beneficial for examining the relationships between estimated evolutionary events and other biological features.

In this study, to test the constancy of the protein evolutionary rate in the evolution of closely related species, we examined changes in protein evolutionary rates among the lineages of four species of *Saccharomyces *sensu stricto group: *S. cerevisiae*, *S. paradoxus*, *S. mikatae *and *S. bayanus*. To reveal the biological features of the genes experiencing changes in rate of protein evolution, we compared some evolutionary and functional data, such as molecular function, gene length, expression level and codon usage bias, across four groups of genes: (i) genes evolving at a constant rate (constant-rate genes); (ii) genes experiencing evolutionary rate changes (either acceleration or deceleration: variable-rate genes); (iii) genes experiencing acceleration (rate-accelerated genes); and (iv) genes experiencing deceleration (rate-decelerated genes).

## Results & discussion

### Estimation of the number of genes with changes in protein evolutionary rates

**Figure 1:**
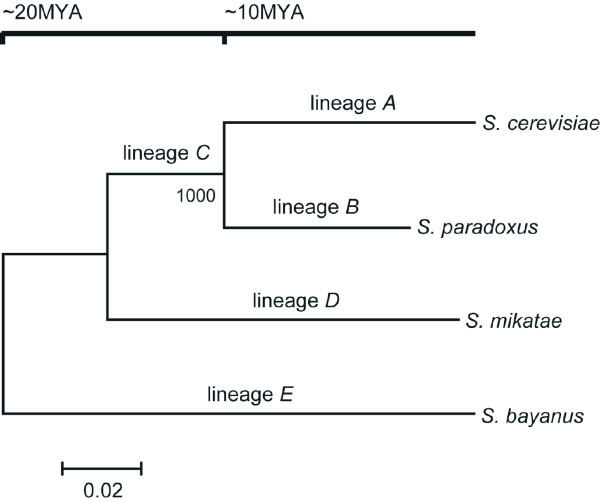
**Phylogenetic relationships of the four species of the *Saccharomyces *sensu stricto group**. The phylogenetic tree was constructed with the number of nucleotide substitutions, estimated by Kimura's two-parameter method, of concatenated nucleotide sequences of 2,610 orthologous genes (3,776,106 sites) by the neighbor-joining (NJ) method [22]. The names of branches defined in this study are shown above each branch. The number below the interior branch represents the bootstrap value (1000 replicates).

We constructed 2,610 unambiguous 1:1:1:1 orthologous genes among four closely related sensu stricto *Saccharomyces *species (*S. cerevisiae*, *S. paradoxus*, *S. mikatae *and *S. bayanus*) by using synteny-based orthology information and data cleaning processes (see Methods). Figure 1 shows the phylogenetic tree which was constructed by the neighbor-joining (NJ) method [22] based on the concatenated nucleotide open reading frame (ORF) sequences of 2,610 orthologous genes (3,776,106 sites), which was consistent with the consensus phylogeny [23]. The genetic distances between species were based on the numbers of nucleotide substitutions estimated by Kimura's two-parameter method [24]. In further analysis, we used this tree topology as the standard phylogenetic relationship among four species of *Saccharomyces *sensu stricto group.

For each of the 2,610 orthologous genes, the number of synonymous substitutions per synonymous site (*dS*) and the number of nonsynonymous substitutions per nonsynonymous site (*dN*) in the five lineages shown in Figure 1 was estimated by the maximum-likelihood method implemented in the PAML CODEML program [25]. In Table 1, medians, means and standard errors of *dS *and *dN *values for each lineage are shown. Both *dS *and *dN *values in the lineage *B *are significantly lower than those in the lineage *A *(Wilcoxon rank sum test; *P *< 2.2 × 10^-16^). This trend could be also observed as a shorter branch length of the lineage *B *when compared with the lineage *A *in Figure 1. The lower rates of substitutions in the lineage leading to *S. paradoxus *would reflect a lower mutation rate in this lineage [19]. This species-specific lower mutation rate would not cause any change in the *dN*/*dS *ratio across the lineages, because changes in mutation rates should affect both *dS *and *dN *in the same way.

**Table 1:** Evolutionary rates (*dS*,*dN *and *dN*/*dS*) of 2,610 orthologous genes for each lineage

	*dS*			*dN*		
	
Lineage	Median	Mean	S.E.	Median	Mean	S.E.
*A*	0.238	0.237	0.001	0.019	0.023	0.000
*B*	0.141	0.147	0.005	0.011	0.014	0.000
*C*	0.147	0.150	0.001	0.012	0.014	0.000
*D*	0.352	0.355	0.002	0.029	0.034	0.001
*E*	0.695	0.698	0.005	0.057	0.064	0.001

The mean, median and S.E. of *dN*/*dS *value of all lineage based on the one-ratio model are 0.081, 0.094 and 0.001, respectively.

**Figure 2:**
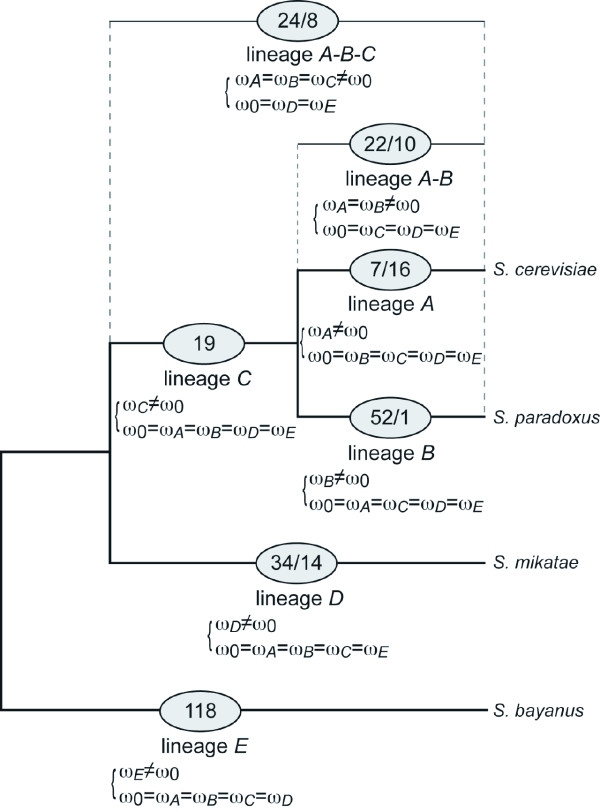
**The evolutionary models tested in the likelihood ratio tests and the numbers of the genes with rate changes, rate accelerations and rate decelerations for each lineage**. Evolutionary models in which ω (*dN*/*dS *ratio) on a certain lineage (ω_*x*_) assumed to be different from ω on other background lineages (ω_0_) are shown below each branch. ω_*x *_is the nonsynonymous/synonymous substitution rate ratio in the lineage *X*. The numbers of the genes with evolutionary rate accelerations and rate decelerations estimated by the likelihood ratio tests are shown in the circles drawn on each branch. For the lineage *C *and *E*, the numbers of the genes with evolutionary rate changes are shown.

To examine the constancy of protein evolutionary rates in the four *Saccharomyces *species, we performed a series of likelihood ratio tests (LRTs) between constant-rate and variable-rate models for each orthologous gene (see Methods). The results of the LRTs showed that the *dN*/*dS *ratios of 344 (13.2%) genes have changed significantly, in at least one lineage, during the evolution of the four species of the *Saccharomyces *sensu stricto group (*P *< 0.05, after correction of multiple tests by Bonferroni-correction). As shown in Figure 2, based on the maximum-parsimony principle, we found that the rates of protein evolution of 7, 52, 22, 24 and 34 genes were accelerated in the lineages *A*, *B*, *A-B*, *A-B-C *and *D*, respectively. Similarly, the protein evolutionary rates of 16, 1, 10, 8 and 14 genes were decelerated for the corresponding lineages. The *dN*/*dS *ratios of 19 and 118 genes have been changed in the lineages *C *and *E*, respectively. Because these rate changes could not be explained by one rate-change event (either rate-acceleration or rate-deceleration event) and there is no outgroup lineage, we could not easily distinguish rate acceleration from rate deceleration. In addition, the rates of protein evolution of 19 genes differ from lineage to lineage. Overall, the protein evolutionary rates of at least 139 genes were accelerated, whereas those of 49 genes were decelerated. These numbers are in a proportion of 2.8:1, suggesting that accelerated evolution was about three times more frequent than decelerated evolution in the evolution of four *Saccharomyces *species. However, the proportion of these two types of genes varied from lineage to lineage, and the ratio of number of the genes with rate accelerations to those with rate decelerations was significantly lower in the lineage *A*, and higher in the lineage *B*, than for the average of all lineages (Fisher's exact probability test; *P *< 0.001). These observations may reflect some species-specific biological features, such as changes in the growth environment, in the lineages *A *and *B*. Yang (1998) [26] reported that, by the LRT, the significant variations in *dN*/*dS *were observed at 22 of 48 nuclear loci across the lineages of primates, artiodactyls and rodents. However, these results are not easily comparable with ours because they examined the rate variations over a longer evolutionary time span (divergence time among these species is approximately 90 MYA) and analyzed a limited number of genes. Therefore, our results would be expected to show more reliable estimates of changes in protein evolutionary rates at the genome-wide level.

To confirm the changes in *dN*/*dS *ratio estimated by the LRTs, we tested the differences in the ratio of number of nonsynonymous to synonymous substitutions, estimated by the approximate method performed by yn00 in the PAML package, between the lineage of interest and other lineages by Fisher's exact probability test. Most of the rate changes (82.6%) were confirmed by this test (*P *< 0.05). Because the maximum-likelihood method considers a more realistic evolutionary model and has some advantages [26,27], compared with the approximate method, we analyzed the genes experiencing changes in protein evolutionary rates estimated by the LRTs in this study.

Although the number of genes with changes in protein evolutionary rates differs from lineage to lineage, we roughly estimated the frequency of the rate changes in the course of closely related *Saccharomyces *evolution. In order to estimate the evolutionary time scales for each lineage, we constructed the linearized tree based on the concatenated nucleotide sequences of the genes which evolved at constant rates. As shown in Figure 1, the divergence time between *S. cerevisiae *and *S. paradoxus *was assumed to be 10 MYA and that between *S. bayanus *and the common ancestor of the other three species was assumed to be 20 MYA. The results show that the changes in rates of protein evolution have occurred with a probability of 2.0 × 10^-3 ^per gene per million years on average. Furthermore, when we divided it into the rate-acceleration and the rate-deceleration, each frequency was at least 1.3 × 10^-3 ^and 4.6 × 10^-4 ^per gene per million years, respectively. In this study, because we assumed no variations in *dN*/*dS *ratio among sites and then averaged the ratio over all sites in the gene, we could only detect the variations in the rate of protein evolution that occurred over a large part of a given gene in a certain lineage. Thus, it should be noted that we may have missed the rate changes occurring over a small part of the gene and underestimated the number of genes with rate changes.

### Most of the changes in rates of protein evolution would be caused by changes in the strength of functional constraint

There are two possible explanations for the changes in rates of protein evolution. One is positive Darwinian selection, and the other is change in the strength of functional constraints. It is generally thought that the genes which experienced strong positive Darwinian selection will have a *dN*/*dS *ratio greater than one. Thus, to examine the contribution of positive Darwinian selection to the changes in rates of protein evolution, we searched for the rate-accelerated genes that have a *dN*/*dS *ratio greater than one in the lineage in which rate acceleration occurred. The *dN*/*dS *ratios for each lineage were estimated by the CODEML program under the free-ratio model. As a result, we found only six genes in which accelerated evolution might be driven by positive Darwinian selection (Table 2). It is difficult to explain the rate decelerations by positive Darwinian selection, because positive selection usually acts to increase a *dN*/*dS *ratio in a given lineage. Therefore, this result suggested that the remaining changes in protein evolutionary rates (338 of 344 genes) were mainly caused by changes in the strength of functional constraints during their evolution. Because positive Darwinian selection often acts on a small number of amino acid sites in a short period of evolutionary time, it is difficult to detect it and such type of adaptive evolution might be the cause of rate-acceleration in some of the genes.

**Table 2:** The genes in which accelerated evolution might be driven by positive Darwinian selection

**Gene ID**	**Description**	**Lineage**^a^	***dN/dS***				
			
			***A***	***B***	***C***	***D***	***E***
YDR197W	Mitochondrial translational activator of the COB mRNA	*B*	0.078	**1.472**	0.092	0.088	0.131
YER174C	Hydroperoxide and superoxide- radical responsive glutathione- dependent oxidoreductase	*B*	0.086	**1.243**	0.033	0.102	0.044
YFR041C	telomere maintenance	*B*	0.132	**1.138**	0.054	0.111	0.071
YGL119W	Protein required for ubiquinone (coenzyme Q) biosynthesis and for respiratory growth	*B*	0.045	**4.607**	0.044	0.073	0.078
YMR006C	glycerophospholipid metabolism	*C*	0.060	0.036	**2.788**	0.060	0.052
YPL248C	galactose metabolism; DNA- dependent regulation of transcription	*C*	0.175	0.240	**1.078**	0.145	0.170

^a^: Lineage in which the accelerations of protein evolutionary rates occurred.

Changes in the *dN*/*dS *ratio can be caused by changes in selective pressure acting on either synonymous or nonsynonymous sites. The major selective pressure acting on synonymous sites is codon usage bias. It is generally thought that codon preferences reflect a balance between mutational biases and natural selection for translational optimization and that optimal codons help to achieve faster translation rates and high accuracy [28]. Using the codon adaptation index (CAI) [29] as a measure of codon usage bias, we examined whether the changes in *dN*/*dS *ratio were due to differences in codon usage bias among the species. First, to examine whether codon usage biases acting on the genes with rate changes were more varied than those on the genes with constant rate among the four *Saccharomyces *species, we calculated the coefficient of variation of CAI among the four species and compared the distributions of those between the two groups of genes. The resulting means of the coefficient of variation of CAI were 0.054 and 0.051 for the genes with constant rates and rate changes, respectively, and therefore were not very different. Rather, in the constant-rate genes, codon usage bias was more varied across the species. Next, for each gene with rate changes, we examined whether the changes in *dN*/*dS *ratio can be explained by higher or lower codon usage bias in a specific lineage. For detecting the lineages in which the codon usage bias was higher or lower than others, we set the cut-off of CAI at the mean plus or minus the standard deviation. Under this criterion, 2,541 (97.4%) genes showed variations in CAI across the four species. As a result of detailed analysis of the relationships between patterns of the changes in *dN*/*dS *ratio and the variations of codon usage bias for each gene, we found that only nine rate changes could be explained by changes in codon usage bias in the specific lineage; these genes were *YDR490C*, *YEL036C*, *YER174C*, *YGL141W*, *YIR003W*, *YJL061W*, *YLR298C*, *YMR301C *and *YPR113W*. For example, *YEL036C*, for which accelerated evolution was observed in the lineage *D*, had stronger codon usage bias in *S. mikatae *than in other species. This stronger codon usage bias acting on the synonymous sites in the lineage of *S. mikatae *might have caused the apparently higher *dN*/*dS *ratio of *YEL036C *in the lineage *D*. These results suggest that, although nine potential cases of variations in selective pressures acting on synonymous sites were observed, this mechanism was not the major reason for the changes in rates of protein evolution. Therefore, most of the changes in protein evolutionary rates would be caused by the changes in functional constraints acting on the nonsynonymous sites.

### Contribution of paralogous gene duplication and loss to the changes in rates of protein evolution

In 1970, Ohno proposed that an increase in rates of protein evolution occurs immediately after gene duplication, because of relaxation of the functional constraints acting on the genes involved in the event [10]. Because we excluded from our analysis orthologous genes that have experienced recent species-specific duplication and loss by the process of estimation of 1:1:1:1 unambiguously defined orthologous genes, none of the rate changes seemed to be caused by recent gene duplication and loss. However, when paralogous genes, which were duplicated before the divergence of the four *Saccharomyces *species, of a given orthologous gene have similar functions and compensate for the function of each other, a species-specific paralogous gene duplication and loss may affect the functional constraints acting on the orthologous gene, and thus may cause the changes in protein evolutionary rates in a given lineage. To examine this possibility, we estimated the paralogous gene duplication and loss that occurred in the course of the evolution of four *Saccharomyces *species by checking the variations in the number of family members among the four species (see Methods). As a result of detailed investigations of the relationships between changes in protein evolutionary rates and paralogous gene duplications and losses for each gene with rate changes, we found only one case (*YGL255W*) in which the rate change could be explained by loss of a paralogous gene. In this case, the strengthened functional constraints in the lineage *D *due to the loss of a paralogous gene might be the cause of the protein evolutionary rate deceleration in the lineage *D*. This suggests that most of the changes in rates of protein evolution due to the changes in functional constraints would have occurred in the evolution of *Saccharomyces *species, independently of gene duplications and losses.

### Biological function of the genes with protein evolutionary rate changes

To reveal biological functions of the genes with the changes in protein evolutionary rates, we used the Gene Ontology (GO) annotation. GO terms assigned to each gene in the *S. cerevisiae *genome were obtained from SGD ftp site and statistical significances were assessed by using GO::TermFinder [30] with 5% false discovery rate cut-off level. As a result, "the tricarboxylic acid (TCA) cycle" related terms ("succinate dehydrogenase complex (ubiquinone)", "respiratory chain complex II", "succinate dehydrogenase complex" and "fumarate reductase complex") were overrepresented in the genes with rate-change. Under the presence of glucose, *Saccharomyces *generally prefer to metabolize glucose by using the fermentative Embden-Meyerhof pathway and produce ethanol even when oxygen is abundant. When the glucose is exhausted, cells undergo a "diauxic shift", in which they switch to a fully respiratory metabolism, catabolizing carbon compounds via the TCA cycle and oxidative phosphorylation in the mitochondria. Indeed, under conditions of glucose scarcity, the expression levels of TCA cycle related genes were significantly increased compared with those under abundant glucose conditions [31]. Therefore, the functional constraints acting on these genes might be highly variable by the effect of growing environment during the *Saccharomyces *evolution. Furthermore, "transport between nucleus and cytoplasm" and "nuclear pore organization" related terms ("snRNP protein import into nucleus", "ribosomal protein import into nucleus", "snRNA transport", "tRNA export from nucleus", "rRNA transport", "tRNA transport" and "nuclear pore organization and biogenesis") were significantly enriched in the genes with rate-acceleration in the Lineage *B*. Indeed, one of nuclear pore protein genes, *Nup96*, is known as a gene that causes epistatic inviability in hybrids between two *Drosophila *sibling species, *Drosophila melanogaster *and *D. simulans *[32]. These accelerated evolution of the genes related to "transport" and "nuclear pore organization" might be involved in the speciation between *S. cerevisiae and S. paradoxus*. For other lineages, there are no critical functional biases that characteristic to the genes with rate changes, suggesting that the changes in functional constraints acting on those genes might be caused by a variety of reasons in each lineage. The list of the functional descriptions for all the genes with rate changes is available as an additional file (Additional File 1).

### Biological features of the genes with changes in protein evolutionary rates

To reveal biological features of the genes with rate changes, we compared some evolutionary and functional data, such as gene length, mRNA expression level and codon usage bias, across four groups of genes consisting of the genes with constant rates, variable rates, rate accelerations and rate decelerations.

First, the lengths of nucleotide ORF sequences were measured in *S. cerevisiae*, because almost all *S. cerevisiae *genes have been experimentally validated, and thus are more reliable than those measured in other species. The gene lengths (mean ± standard error) were 1,460 ± 18 bp and 1,731 ± 53 bp for the constant-rate and variable-rate genes, respectively, and the variable-rate genes have significantly longer gene lengths than the constant-rate genes (Wilcoxon rank sum test; *P *= 2.1 × 10^-8^). In addition, the mean gene length for the genes with rate accelerations was 1,731 ± 78 bp, and for those with rate decelerations was 1,508 ± 133 bp. The rate-accelerated genes have significantly longer gene lengths than the constant-rate genes (Wilcoxon rank sum test; *P *= 2.5 × 10^-4^) and also longer than the rate-decelerated genes but not significantly. Longer gene lengths of the rate-accelerated genes suggested that a longer sequence might be required for accumulation of still more substitutions. In other words, because a large fraction of shorter protein-coding genes consists of functionally important sequences, the protein evolutionary rates of shorter genes might be difficult to accelerate.

**Figure 3:**
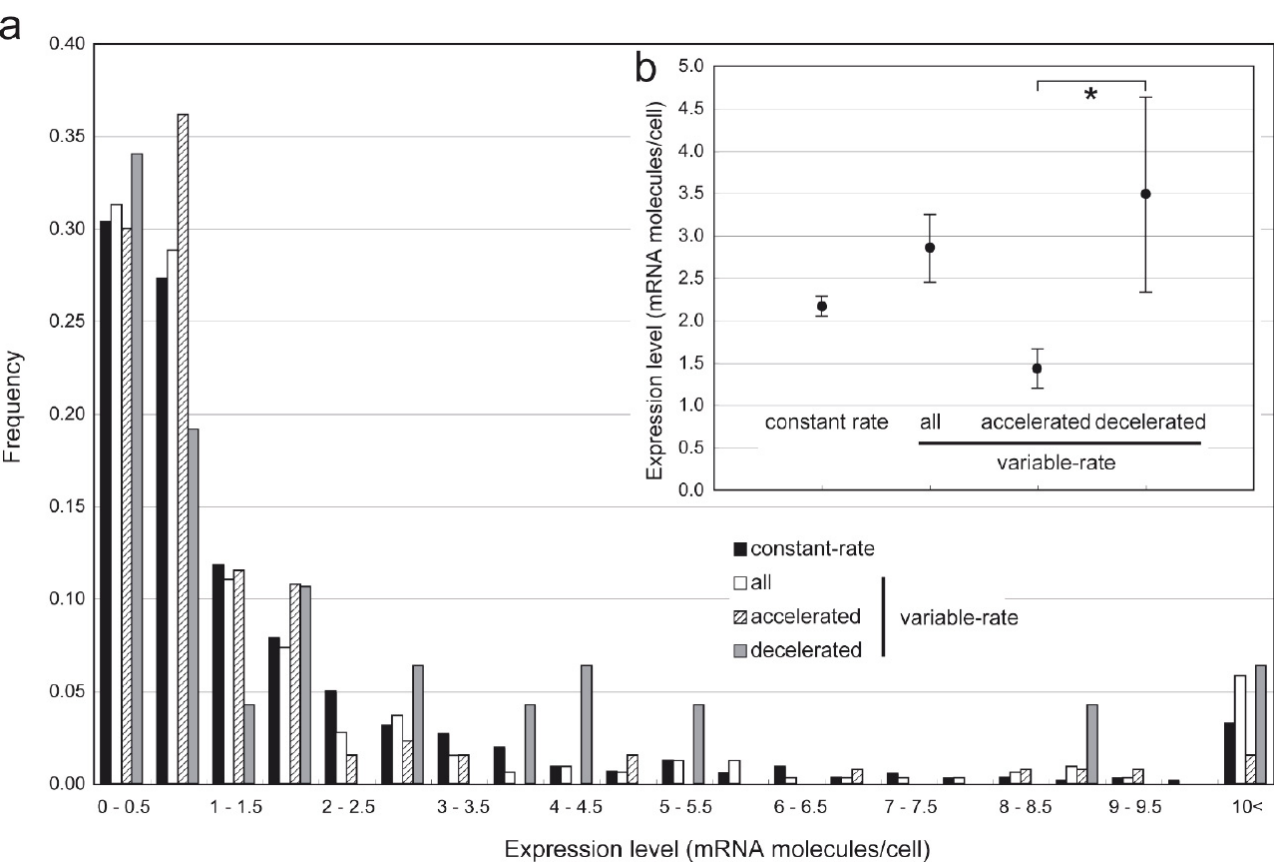
**Relationship between the changes in rate of protein evolution and mRNA expression levels**. (a) Histogram showing the distribution of mRNA expression levels (mRNA molecules/cell) for the genes with constant evolutionary rates and the three groups of the genes with evolutionary rate changes. (b) The means of mRNA expression levels with standard errors are shown. Statistically significant differences in mRNA expression level between rate-accelerated and rate-decelerated genes are indicated by an asterisk (*: *P *< 0.05), when we limited the rate variations to those occurred in the lineages leading to *S. cerevisiae *after the divergence from *S. mikatae*.

In order to examine the relationships between the mRNA expression levels and the changes in rate of protein evolution during evolution, we used two kinds of mRNA abundance data (mRNA molecules/cell) measured by high-density oligonucleotide array [20] and DNA microarray [21]. As shown in Figure 3, for the high-density oligonucleotide array data of Holstage et al. (1998) [20], no significant difference in the expression level was observed between the constant-rate and variable-rate genes; the mean mRNA abundances were 2.17 ± 0.12 and 2.85 ± 0.40 mRNA molecules/cell, respectively. Furthermore, the mRNA abundances of the rate-accelerated genes were lower than those of the rate-decelerated genes, though the difference is not significant; the mean mRNA abundances were 1.47 ± 0.22 and 3.19 ± 0.99 mRNA molecules/cell, respectively. When we used the DNA microarray data of Wang et al. (2002) [21], similar results were observed (data not shown).

**Figure 4:**
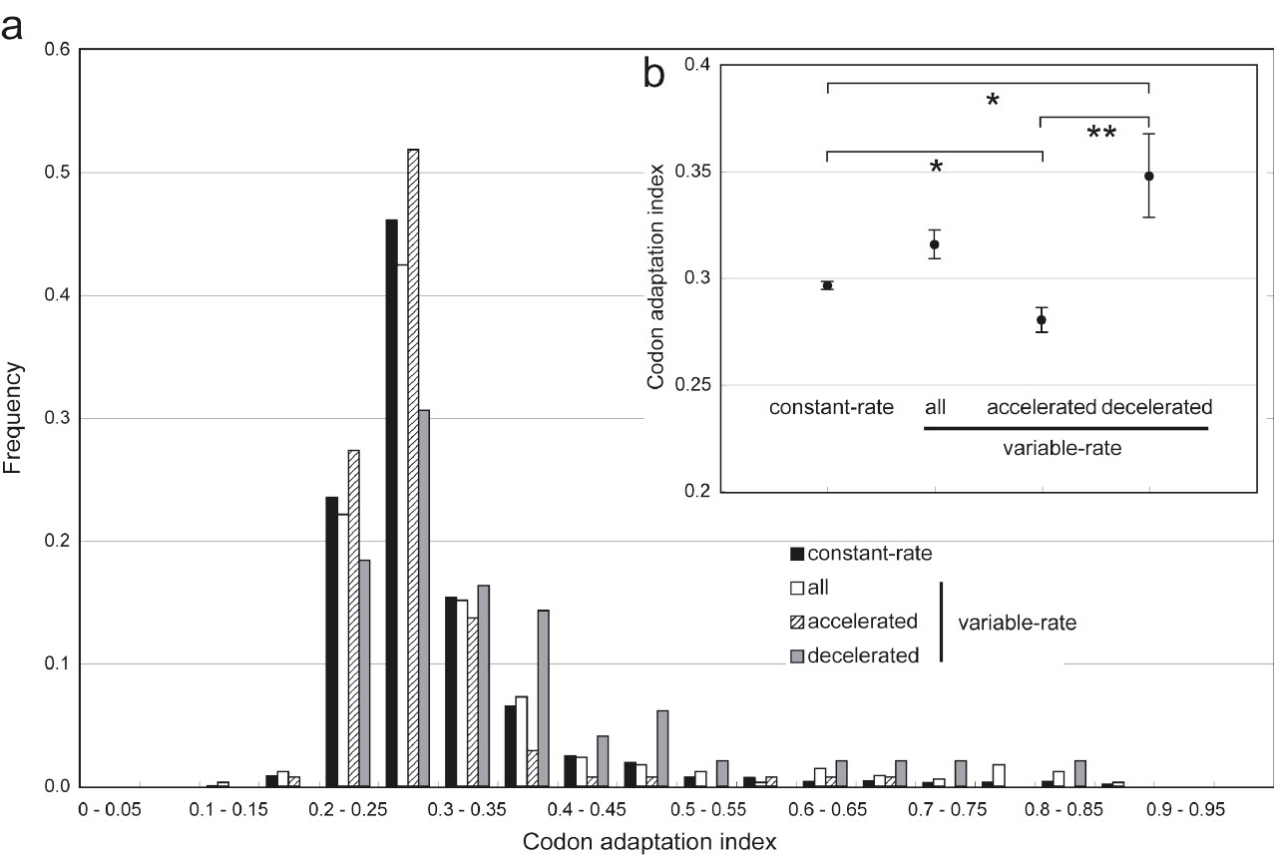
**Relationship between the variations in rate of protein evolution and codon adaptation index**. (a) Histogram showing the distribution of codon adaptation index for the genes with constant evolutionary rates and the three groups of the genes with evolutionary rate changes. (b) The means of codon adaptation index with standard errors were shown. Statistically significant differences in codon adaptation index between groups are indicated by asterisks (*: *P *< 0.05, **: *P *< 0.01)

To further examine the relationships to the expression levels, codon usage bias was used as the second measure of mRNA expression level. Codon usage bias is more likely to reflect the level of expression linked to protein evolution, because it determines the efficiency of translation and therefore will be a good predictor of expression level over the evolutionary history of the gene, rather than at a single time point in the laboratory. We used mean CAI among the four species as a measure of codon usage bias for each gene. As shown in Figure 4, a significant difference in CAI was observed between the rate-accelerated and -decelerated genes (Wilcoxon rank sum test; *P *< 0.01); the mean CAIs were 0.28 ± 0.01 and 0.34 ± 0.02, respectively. The genes with rate accelerations have significantly lower CAI and those with rate decelerations have significantly higher CAIs than the constant-rate genes (Wilcoxon rank sum test; *P *< 0.05); the mean CAI of the constant-rate genes was 0.30 ± 0.00. Together with the results from analysis using mRNA abundance data, these results suggest that the rate-accelerated genes have lower expression levels, and have weaker codon usage biases, than the constant-rate and rate-decelerated genes. When we used effective number of codons as another measure of codon usage bias, we also obtained essentially the same results (data not shown).

Some of the aforementioned functional data, such as gene length and mRNA abundance, were measured only in *S. cerevisiae*. Therefore, we limited the rate changes to those occurring in the lineages leading to *S. cerevisiae *after the divergence from the *S. mikatae *lineage, that is the lineages *A*, *A-B *and *A-B-C*, and performed the same analysis as described above. In the limited data, the numbers of the genes with rate accelerations and decelerations were reduced to 53 and 34 genes, respectively. As a result, the genes with rate accelerations still showed significantly lower expression levels (Wilcoxon rank sum test; *P *< 0.05) than those with rate decelerations. The significantly lower levels of CAI for the genes with rate accelerations, corresponding to weaker codon usage bias, were also confirmed in the limited data. However, although the genes with rate accelerations have gene lengths even longer than those with rate decelerations (the mean ± standard error gene lengths were 1,627 ± 133 and 1,586 ± 205 bp, respectively), no significant difference was observed between the two groups of genes. Thus, the gene length may seem to weakly relate to the variability of the rate of protein evolution. Taken together, these results suggest that the probability of the acceleration of protein evolutionary rates was highly influenced by the selection underlying level of mRNA expression. It is thought that this is due to an excessive accumulation of nucleotide substitutions that would be a significant problem for highly expressed genes.

Recent studies have reported that selection for translational efficiency and accuracy (translational selection) was a major cause of the variation in protein evolutionary rate among genes in the Yeast genome [33], and that the protein evolutionary rate was negatively correlated to mRNA abundance in the cell and codon usage bias. Indeed, in our dataset, mean *dN*/*dS *ratios among the four species were negatively correlated to the mRNA abundances (n = 2481, *P *= 4.5 × 10^-73^, Spearman's rank correlation coefficient: *r *= -0.35) and CAI (n = 2481, *P *= 6.3 × 10^-210^, Spearman's rank correlation coefficient: *r *= -0.57). From these results, we concluded that the protein evolutionary rates of the genes having relatively high *dN*/*dS *ratios, lower expression levels and weaker codon usage biases tend to be accelerated during the evolution of closely related species and this directional selection may increase variation of protein evolutionary rates.

## Conclusion

Using the LRTs, we found that the rates of protein evolution of many genes changed even in the evolutionary course of closely related biologically and morphologically similar species. Furthermore, we found that the genes with rate accelerations tend to have lower expression level and weaker codon usage bias than those with rate decelerations, suggesting that selection for translational efficiency and accuracy may underlie the variability of the protein evolutionary rates. Therefore, when we estimate the accurate evolutionary times or phylogenetic relationships of species based on the sequence divergences of orthologous genes, we should be careful about variable-rate genes, because amino acid substitutions did not occur in proportion to evolutionary time in such genes. In addition, it is also necessary to be very careful to regard such genes as biologically and evolutionally meaningful, because such rate-changes possibly occur frequently even in the evolution of closely related species. Although it is still not clearly understood why such changes in selective constraints acting on the genes occurred, our research sheds light on the features of dynamic changes in protein evolutionary rates in the evolution of closely related species and on the major factors for the rate variability at the genome-wide level.

## Methods

### Construction of the set of 1:1:1:1 orthologous genes among four *Saccharomyces *sensu stricto species

The entire set of nucleotide ORF sequences of *S. cereßvisiae *were downloaded from the Saccharomyces Genome Database (SGD, updated on 26 Jan, 2006) [34]. For *S. paradoxus*, *S. mikatae *and *S. bayanus*, the nucleotide ORF sequences predicted by Kellis et al. (2003) [19] were downloaded from SGD (updated on 15 Dec, 2004). The numbers of downloaded nucleotide ORF sequences were 5,874, 8,955, 9,057 and 9,424, respectively. All nucleotide ORF sequences were translated into amino acid sequences, and we excluded truncated ORF sequences from the dataset. As a result of this process, the numbers of ORF sequences were reduced to 5,855, 7,474, 7,095 and 7,948, respectively. To extract 1:1 orthologous genes between *S. cerevisiae *and other species, using these ORF datasets and the information of potential orthologous gene sets estimated by synteny-based orthology designation [19], we discarded the genes of other three species that corresponded to two or more *S. cerevisiae *genes. The information whether a given gene hits a single *S. cerevisiae *gene or multiple genes is contained in the ORF sequences files downloaded from SGD. As a result, 3,622 sets of orthologous genes among the four *Saccharomyces *species were obtained. Next, we aligned the translated amino acid ORF sequences by ClustalW [35] with the default parameters, and the aligned amino acid sequences were reverse-translated into nucleotide sequences, converting a single gap into a group of three gaps using in-house perl scripts.

For each orthologous gene, the number of synonymous substitutions per synonymous site and the number of nonsynonymous substitutions per nonsynonymous site in each lineage were estimated by the maximum-likelihood method implemented in the PAML CODEML program [25]. To determine equilibrium codon frequencies, we used two models. The first model used the nucleotide frequencies at the three positions within the codon and had 9 = 3 × (4 - 1) parameters. The second model used empirical estimates of 61 codon frequencies and had 60 parameters. Likelihood ratio tests comparing these two models with 51 (= 60 - 9) degrees of freedom suggested statistically significant differences for 2,625 of 3,622 (72.5%) orthologous genes, and the second model was better in the majority of cases. *dN *and *dS *estimated with both models were similar and results obtained using empirical estimates of codon frequencies were used in further analysis.

To avoid contamination of paralogous genes and underestimation of the numbers of synonymous substitutions due to the saturation of synonymous sites, we excluded the genes having a *dS *greater than 1.0 in at least one lineage. We also excluded the genes having less than one synonymous substitution in at least one lineage. Finally, we obtained 2,610 unambiguously defined 1:1:1:1 orthologous genes among the four closely related *Saccharomyces *species.

### Statistical test for detecting changes in protein evolutionary rate across lineages by likelihood ratio tests

To test the constancy of rate of protein evolution during the evolution of four *Saccharomyces *species, we performed a series of likelihood ratio tests between constant-rate and variable-rate models for five lineages corresponding to four exterior branches and one interior branch shown in Figure 1[26,36]. We used the nonsynonymous/synonymous substitution rate ratio (denoted as *dN/dS *ratio or ω) as a measure of protein evolutionary rate.

First, to test whether rates of protein evolution are different among all evolutionary lineages, we performed the LRT comparing the free-ratio model where all five lineages have different values of ω estimated from the data with the one-ratio model where all lineages share a common value of ω. Because the free-ratio model has five parameters for ω and the one-ratio model has only one, the LRT statistic is calculated as 2 times the differences in maximum log likelihood (2ΔlnL) and is asymptotically distributed as a χ^2 ^distribution with 4 degrees of freedom. Next, we tested whether the lineages of interest have a different ω from other lineages, in other words, whether the protein evolutionary rate has changed at a certain point in the course of evolution. For this purpose, we conducted the LRTs independently, comparing the one-ratio model with seven two-ratio models (Figure 2), which assumes that the lineages of interest have a ω_X _that is different from the background ratio ω_0_. In these LRTs, 2ΔlnL between one-ratio and two-ratio models should follow a χ^2 ^distribution with 1 degree of freedom. In each two-ratio model, we assumed that lineage *A*, *B*, *C*, *D*, *E*, the pair of the lineages *A *and *B *(lineage *A-B*), and the combination of the lineages *A*, *B *and *C *(lineage *A-B-C*) have their corresponding individual ω_X_, respectively. For example, in the case of detection of variation in the rate of protein evolution in the lineage *A*, we assume that the lineage *A *has a certain ω_*A *_that is different from that background (lineage *B*, *C*, *D *and *E*) ratio ω_0 _and compare log likelihood value between one-ratio and this two-ratio models. If the null hypothesis in which ω is constant among the five lineages is correct, 2ΔlnL between the two models asymptotically has a χ^2 ^distribution with 1 degree of freedom.

When the variable-rate (free-ratio and two-ratio) model fit the data significantly better than the constant-rate model, we assigned the variable-rate model as the best-fit evolutionary model of the gene (*P *< 0.05, after correction of multiple tests by Bonferroni-correction). When two or more variable-rate models fit the data significantly, we chose the model with the lowest *P*-value for LRT as the best-fit evolutionary model of the gene. The 2ΔlnL scores and *P*-values under the best-fit evolutionary model for all genes experiencing change in protein evolutionary rate are shown in Additional File 1. Furthermore, for the genes having two different *dN*/*dS *ratios in their evolution, we assigned the lineage and the direction of change in the *dN*/*dS *ratio (acceleration or deceleration of protein evolutionary rate) based on the maximum-parsimony principle.

To confirm the genes experiencing rate changes estimated by LRTs, we compared the ratio of the number of nonsynonymous to synonymous nucleotide substitutions in the lineage of interest with the average ratio in other lineages by Fisher's exact probability test. The ancestral nucleotide sequences of two interior nodes were estimated by the joint reconstruction method implemented in PAML [37], and estimation of the numbers of synonymous and nonsynonymous substitutions were performed with the yn00 program in PAML package [27].

### Identification of duplicated genes and estimation of paralogous gene duplication and loss

To identify duplicated genes in each of the four *Saccharomyces *genomes, every translated amino acid ORF sequence was used as a query to search against all other ORF sequences using the SSEARCH program in FASTA package [38]. In this analysis, we excluded the ORF sequences that were estimated as one-to-one orthologous genes of *S. cerevisiae *dubious ORF by synteny-based orthology designation [19] from the ORF sequences for *S. paradoxus*, *S. mikatae *and *S. bayanus*. Duplicated genes in each genome were identified as hits with an E-value of smaller than 10 that could be aligned over 50% of the length of the longer protein, following the study of Gu et al. (2003) [39]. In addition, we also used a cutoff threshold of identity score between two ORFs (sequence identity >30% in amino acids), according to the study of Gu et al. (2002) [40]. As a result of this analysis, we obtained 1,930, 1,776, 1,508 and 1,553 duplicated genes for *S. cerevisiae*, *S. paradoxus*, *S. mikatae *and *S. bayanus*, respectively. We estimated the number of gene families and the members of each family by a single linkage clustering of duplicated genes for each species. Furthermore, for each orthologous gene, based on the phylogenetic relationships and the numbers of family members for each species, we estimated species-specific paralogous gene, which were duplicated before the divergence of the four species, duplication and loss occurring in the evolution of the four *Saccharomyces *species. When three species, for example *S. paradoxus*, *S. mikatae *and *S. bayanus*, have five members in a certain gene family, whereas only four genes in the corresponding *S. cerevisiae *gene family, we can estimate a paralogous gene loss in the lineage leading to *S. cerevisiae *(lineage *A*) based on the maximum-parsimony principle.

### Functional genomic data

As measurements of expression levels, we used two sets of mRNA abundance data measured by high-density oligonucleotide arrays [20] and cDNA microarrays [21]. As measurements of expression levels and selective constraints acting on synonymous sites, we used the codon adaptation index (CAI) [29] and the effective number of codons (ENC) [41]. The set of optimal codons for calculating CAI values was taken from the top 20 highly expressed genes in *S. cerevisiae *[42].

## Authors' contributions

YK designed and carried out the study, drafted the manuscript. TI participated in its coordination and helped to draft the manuscript. Both authors read and approved the final manuscript.

## Supplementary Material

Additional File 1 Functional descriptions and log-likelihood differences between constant-rate and variable-rate models for each gene with rate changes. Click here for file
